# Genetic Analysis of Stayability and its Relationships with Production, Conformation, Fertility and Health Traits in Holstein Cattle

**DOI:** 10.3390/vetsci12111105

**Published:** 2025-11-19

**Authors:** Honghong Hu, Zhaodi Xu, Liyun Han, Zhixuan Qiao, Yi Wang, Yikun Jia, Tong Mu, Yun Ma

**Affiliations:** 1 School of Life Science, Yan’an University, Yan’an 716000, China; h918622@163.com (H.H.); 18303418951@163.com (Z.Q.); w3522315196@163.com (Y.W.); 18590078520@163.com (Y.J.); 2 College of Animal Science and Technology, Ningxia Key Laboratory of Ruminant Molecular and Cellular Breeding, Ningxia University, Yinchuan 750021, China; 18235431843@163.com; 3 Ningxia Agriculture Reclamation Dairy Co., Ltd., Yinchuan 750021, China; m15296979603@163.com; 4 School of Life Science, Ningxia University, Yinchuan 750021, China

**Keywords:** longevity, heritability, genetic correlation, survival analysis, Chinese Holstein cattle

## Abstract

The objectives of this study were to estimate genetic parameters for stayability in Chinese Holstein cattle and to estimate its genetic correlations with routinely collected traits. Although knowledge and genetic breeding technologies for dairy cattle have advanced, the lifespan of dairy cows has been declining, mainly due to increased production stress and associated health problems. This makes the genetic improvement of Holstein cattle crucial for sustainable production. Stayability is an indicator trait reflecting longevity, measured as the survival probability of dairy cows at specific stages. To evaluate temporal differences in herd retention, we defined 7 stayability traits as the ability of cows to remain in the herd for 36 (S36), 42 (S42), 48 (S48), 54 (S54), 60 (S60), 72 (S72), and 84 (S84) months after first calving. Data from 56,630 cows between 2011 and 2020 across 14 dairy farms in Ningxia were considered for stayability analyses. Survival analysis using non-parametric methods showed that cattle with sterility had the longest survival time, while those with abomasal displacement had the shortest survival time. The heritability estimates (±standard error) for S36, S42, S48, S54, S60, S72 and S84 were 0.048 ± 0.006, 0.063 ± 0.006, 0.074 ± 0.007, 0.099 ± 0.007, 0.115 ± 0.007, 0.088 ± 0.007, 0.118 ± 0.008, respectively. The genetic and phenotypic correlations among stayability traits ranged from 0.382 to 0.975 and from 0.090 to 0.799, respectively. Furthermore, the approximate genetic correlations between stayability and routinely collected traits (8 production, 20 conformation, 13 fertility, 13 health traits) were assessed. Stayability showed low to moderate genetic correlations with most of these traits. In summary, developing a selection index that incorporates stayability is expected to improve the longevity of dairy cows. All unfavorable genetic relationships observed between stayability and other routinely collected traits should be accounted for in a selection index, thereby enhancing the lifespan of Holstein cattle while maintaining or improving productive performance.

## Simple Summary

Stayability refers to the ability of a cow to remain in a herd to a given time point and can serve as an indirect indicator of longevity when culling dates are unavailable or the individual is still alive in the herd. Therefore, accurately evaluating stayability traits and their genetic relationships with conventional traits in breeding objectives is particularly important. This study provides a comprehensive genetic evaluation of stayability in Holstein cattle in Ningxia. Stayability showed low heritability, but high genetic and phenotypic correlations between adjacent stayability traits. In addition, stayability had low to moderate genetic correlations with conventional traits, including production, conformation, fertility, and health traits. These findings help provide new trait reference indicators for improving the breeding of Holstein cattle.

## 1. Introduction

The longevity of dairy cows is a composite indicator that integrates production, reproduction, and health traits [1], and its economic value accounts for approximately 50% of the economic value of production traits. Therefore, selecting for longevity involves the improvement of multiple functional traits and is crucial for the sustainable development of the dairy industry. Since the 1950s, the longevity of dairy cows has been a consistent focus in the global dairy industry [2]. However, due to its low heritability and the fact that phenotypic data are typically recorded late in the cow’s life, the lifespan of dairy cows continues to decline worldwide, with productive life ranging from 2.1 to 4 years [3,4,5].

Stayability is the ability of a cow to remain in the herd at a given time, which is a further trait definition reflecting longevity, considering the probability of survival at specific stages [6]. It is usually represented as a binary trait, where 1 indicates that the animal remains in the group and successfully calves at a specific time, while 0 indicates otherwise. Unlike longevity, stayability provides information on longevity in the early stage [6], and can be used to evaluate the survival status of animals when data such as culling dates are scarce. Therefore, improving stayability can not only extend the lifespan of cattle but also reduce the incidence of involuntary culling, and its evaluation has become increasingly important in dairy cow breeding.

Although the evaluation of stayability can incorporate records of animals removed from the herd for unknown reasons, its heritability is low and it is affected by multiple factors, including production, reproductive performance, and various diseases [7,8]. Therefore, indirect selection for stayability is gaining increasing interest, as the relationships between stayability and other economically important traits are crucial for achieving genetic progress through indirect selection. Few studies have investigated these relationships from different perspectives in other countries, collectively providing valuable insights into their potential applications in breeding programs. For example, Parker et al. [9] found significant correlations between stayability and first milk yield, milk fat yield, with correlation coefficients ranging from 0.176 to 0.195. Hargrove et al. [10] reported a genetic correlation of 0.76 between first milk yield and productive life, while the correlation between first milk yield and lifetime milk yield reached 0.85. This indicates that cows with higher first milk yield tend to exhibit greater lifetime milk production. Hardie et al. [11] found that stayability was positively correlated with milk yield and negatively correlated with fat percent and stillbirth, ranging from 0.1 to 0.18 and −0.17 to −0.42. In addition, Shabalina et al. [6] reported that mastitis and ruminal acidosis at different lactation stages exhibited high negative genetic correlations, reaching up to −0.77 and −0.98, respectively. However, no studies have investigated the relationships between stayability and other traits in China. Therefore, understanding the relationships between stayability, reproductive traits, and health traits is essential for developing targeted breeding strategies and optimizing genetic improvement, ultimately enhancing both production and productive lifespan of dairy herds. The objective of this study were: (1) to analyze reasons for culling in Chinese Holstein cattle, (2) to estimate genetic parameters for stayability traits, (3) to assess approximate genetic correlations between stayability and production, SCS, conformation, fertility, and health traits.

## 2. Materials and Methods

### 2.1. Phenotypic Data

Data from 2011 to 2020 at 14 dairy cattle farms in Ningxia, China. All animals were housed in free-stall housing systems in the same manner, fed TMR, and provided access to water ad libitum. Seven stayability traits were analyzed: the ability of cows to remain 36 months in herd after first calving (S36); the ability of cows to remain 42 months in herd after first calving (S42); the ability of cows to remain 48 months in herd after first calving (S48); the ability of cows to remain 54 months in herd after first calving (S54); the ability of cows to remain 60 months in herd after first calving (S60); the ability of cows to remain 72 months in herd after first calving (S72); the ability of cows to remain 84 months in herd after first calving (S84). All stayability traits are defined as a binary trait (0/1), where a value of 1 indicates that a cow survived to a specific time point after first calving, and a value of 0 indicates that she did not survive to that time point. This definition reflects the proportion of cows surviving to each specific time point. The quality control criteria for traits data were performed as follows: (1) The farms–birth years with fewer than 100 individuals were excluded; (2) The birth-year season with less than 100 individuals were excluded; (3) The age at first calving was categorized into nine levels: ≤23, 24, 25, 26, 27, 28, 29, 30, and >30 months. Further details can be found in the study by Hu et al. [12]. After data editing, the effective observations for S36, S42, S48, S54, S60, S72 and S84 were 56,630, 56,630, 56,630, 56,630, 56,630, 56,630 and 56,630, respectively. The pedigree data contained 133,409 females and 7472 males; each animal was traced to as many ancestors as possible. In addition, it is necessary to standardize the names of the original culling reasons. In this study, the culling reasons were organized and standardized into 9 categories [13,14]. The farm-year of birth, year-season of birth, and age of first calving were significant factors on stayability traits.

### 2.2. Survival Analysis

We used the Kaplan–Meier method to compare survival curves among different diseases, and used the Cox proportional hazards regression model to evaluate the effects of various variables on survival time. The censoring data for surviving individuals was the last date they were recorded in the herd, whereas for deceased individuals, it was the date of death. Survival analysis of the stayability trait was conducted using the R version 4.3.3 package survminer, the model described by Roxstr et al. [15].

### 2.3. Statistical Model

Variance components of stayability traits were estimated using the Average Information Restricted Maximum Likelihood (AI-REML) algorithm implemented in the derivative-free multivariate (DMU) package, based on single-trait and bivariate animal models [16], the model was as follows:

Model 1: Single-trait animal model

y=Xb+Za+e

where y is the vector of phenotype for stayability; b is the vector of fixed effects, including farm-year of birth, year-season of birth, and age of first calving; a is the vector of random additive genetic effects; e is the vector of random residual effects; X, Z are incidence matrices linking phenotypic records to b and a, respectively. The season was divided into four levels: spring (March–May), summer (June–August), autumn (September–November) and winter (December–February), the age of first calving were divided into 9 levels (≤23, 24, 25, 26, 27, 28, 29, 30 and >30 months).

Model 2: Bi-variate animal model

$$\left[\begin{array}{c} y_{1}\\ y_{2} \end{array}\right]=\left[\begin{array}{cc} X_{1} & 0\\ 0 & X_{2} \end{array}\right]\left[\begin{array}{c} b_{1}\\ b_{2} \end{array}\right]+\left[\begin{array}{cc} Z_{1} & 0\\ 0 & Z_{2} \end{array}\right]\left[\begin{array}{c} a_{1}\\ a_{2} \end{array}\right]+\left[\begin{array}{c} e_{1}\\ e_{2} \end{array}\right]$$

where $y_{i}\;$ is the observed value vector of all individuals; $b_{i}\;$ is the fixed effect vector of the *i*th trait including the same fixed effects described for model 1; $\;a_{i\;}$ is the additive genetic effect vector of the *i*th trait; $e_{i}$ is the residual random effect vector of the *i*th trait; $X_{i}$ and $Z_{i}$ are the incidence matrices of $b_{i}$ and $a_{i}$, respectively.

### 2.4. Variance Component

#### 2.4.1. The Formula for Heritability

$$h_{i}^{2}=\frac{\sigma_{a_{i}}^{2}}{\sigma_{P_{i}}^{2}}$$

where $\sigma_{P_{i}}^{2}=\sigma_{a_{i}}^{2}+\sigma_{e_{i}}^{2}$, $\sigma_{a_{i}}^{2}$ is the additive genetic variance, $\sigma_{e_{i}}^{2}$ is the residual variance, $\sigma_{P_{i}}^{2}$ is the phenotypic variance.

#### 2.4.2. The Formula for Genetic Correlation

$$r_{A}=\frac{\mathrm{COV}(a_{1},a_{2})}{\sqrt{\sigma_{a_{1}}^{2}\;\sigma_{a_{2}}^{2}\;}}$$

where $r_{A}$ is the genetic correlation, $\mathrm{COV}(a_{1},a_{2})$ is the additive covariance for $a_{1}$ and $a_{2}$, $\sigma_{a_{1}}^{2}$ is the additive variance for $a_{1}$, $\sigma_{a_{2}}^{2}$ is the additive variance for $a_{2}$.

#### 2.4.3. The Formula for Phenotypic Correlation

$$r_{P}=\frac{\mathrm{COV}(p_{1},p_{2})}{\sqrt{\sigma_{p_{1}}^{2}\;\sigma_{p_{2}}^{2}\;}}$$

where $r_{P\;}$ is the phenotypic correlation, $\mathrm{COV}(p_{1},p_{2})\;$ is the phenotypic covariance for p**_1_**, p**_2_**, $\sigma_{p_{1}}^{2}$ is the phenotypic variance for p_1_, $\sigma_{p_{2}}^{2}$ is the phenotypic variance for p**_2_**.

#### 2.4.4. The SE Formula for the Genetic Correlation


$${\mathrm{SE}}^{2}\left(r\right)=\left[\frac{\sigma_{\mathrm{ij}}^{2}}{{\sigma_{i}^{2}\sigma }_{j}^{2}}\right]\left[\frac{\mathrm{Var}(\sigma_{\mathrm{ij}})}{{\left(\sigma_{\mathrm{ij}}\right)}^{2}}+\frac{\mathrm{Var}({\sigma_{i}}^{2})}{{{4(\sigma }_{i}^{2})}^{2}}+\frac{\mathrm{Var}({\sigma_{j}}^{2})}{{{4(\sigma }_{j}^{2})}^{2}}-\frac{\mathrm{Cov}\left(\sigma_{\mathrm{ij}},{\sigma_{i}}^{2}\right)}{\sigma_{\mathrm{ij}}{\sigma_{i}}^{2}}-\frac{\mathrm{Cov}\left(\sigma_{\mathrm{ij}},{\sigma_{j}}^{2}\right)}{\sigma_{\mathrm{ij}}{\sigma_{j}}^{2}}+\frac{\mathrm{Cov}\left({\sigma_{i}}^{2},{\sigma_{j}}^{2}\right)}{2{\sigma_{i}}^{2}{\sigma_{j}}^{2}}\right]$$

### 2.5. Calculation of Approximate Genetic Correlations

Due to limited access to the raw datasets, the approximate genetic correlations between stayability and routinely collected traits (production, conformation, fertility, health) were calculated through the method proposed by Colo et al. [17], drawing on findings from various previous studies [18,19]. Approximate genetic correlations were calculated based on the estimated breeding values (EBVs) of individuals with reliabilities greater than 0.25 for both traits. The traits included in this analysis are: 8 production traits (milk yield, fat percentage, protein percentage, fat yield, protein yield, lactose percentage, urea nitrogen, SCS); 20 conformation traits (body depth, bone quality, chest width, foot angle, fore attachment, fore teat placement, hoof height, loin strength, median suspensory, rear attachment height, rear attachment width, rear leg rear view, rear legs side view, rear teat placement, rump angle, rump width, stature, teat length, udder depth, angularity; 13 fertility traits and the definition of traits can be found in Hu et al.; 13 health traits (udder health, mastitis, reproductive disorders, gestation disorders and peripartum disorders, irregular estrus cycle and sterility, metritis, digestive disorders, abomasal displacement, metabolic disorders, ketosis, locomotory diseases, claw diseases, laminitis complex. The number of all traits is shown in Supplementary Table S1.

#### 2.5.1. The Formula of Approximate Genetic Correlation

$$r_{g1,2}=\frac{\sqrt{\left(\sum {\mathrm{RL}}_{1}\right)\times \left(\sum {\mathrm{RL}}_{2}\right)}}{\sum \left({\mathrm{RL}}_{1}\times {\mathrm{RL}}_{2}\right)}\times r_{1,2}$$

where $r_{g1,2}$ is the approximate genetic correlation between traits 1 and 2; ΣRL_1_ and ΣRL_2_ are the sums of reliabilities of traits 1 and 2; RL_1_ and RL_2_ are reliabilities of traits 1 and 2; rg_1,2_ is the Pearson correlation between EBV for traits 1 and 2.

#### 2.5.2. The SE Formula for the Approximate Genetic Correlations


$$\mathrm{SE}=\sqrt{\frac{1-r_{g1,2}}{n-2}}$$

## 3. Results

### 3.1. Descriptive Statistics of Stayability Traits

The stayability traits represent the ability of cows to remain in the herd for 36 to 84 months after first calving. Both the number of cows reaching each stayability stage and their survival probabilities declined markedly with increasing months in herd (Table 1), from 13,165 cows at 36 months (0.23) to only 430 cows at 84 months (0.08).

### 3.2. The Survival Analysis for Holstein Cattle

**Figure 1:**
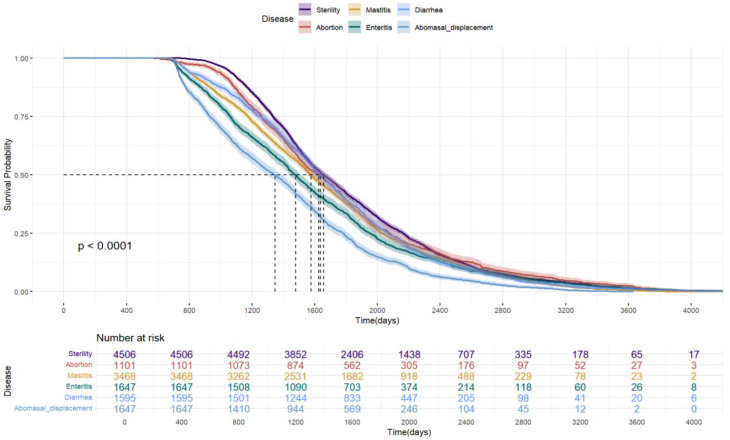
Survival analysis of Holstein cattle.

**Figure 2:**
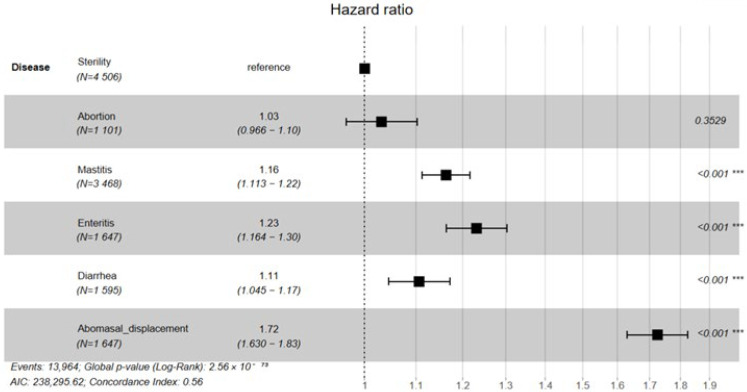
Cox proportional hazards regression analysis of Holstein cattle. Note: *** indicates extremely significant difference.

The Kaplan–Meier survival curves for cows with different diseases are shown in Figure 1. The survival probability declined over time in all disease groups, but the rate of decline differed significantly. The abomasal displacement group showed the fastest decrease and had the shortest median survival time (1350 days), followed by the enteritis and mastitis groups. In contrast, the sterility group exhibited the slowest decline and the longest median survival time (1670 days). Moreover, when sterility was used as the reference category (Figure 2), Cox proportional hazards analysis indicated that mastitis, enteritis, diarrhea, and abomasal displacement all significantly increased the culling risk (*p* < 0.001). Among them, abomasal displacement had the greatest impact, with a culling risk approximately 72% higher than that of the sterility group.

### 3.3. Genetic Parameters of Stayability Traits in Holstein Cattle

The genetic parameters of stayability traits in Holstein cattle are shown in Table 2. The heritability of stayability traits ranged from 0.048 (0.006) to 0.118 (0.008). The genetic correlations among stayability traits were positive, ranging from 0.382 to 0.975, and the phenotypic correlations among stayability traits were positive, ranging from 0.090 to 0.799, respectively. In addition, high genetic and phenotypic correlations were observed between adjacent stayability traits.

### 3.4. Approximate Genetic Correlation Between Stayability and Production Traits

The approximate genetic correlations between stayability and production traits are shown in Table 3. Positive approximate genetic correlations were observed between stayability traits and milk yield, fat yield, protein yield, and lactose percentage, with estimates ranging from 0.180 (S36 and fat yield) to 0.688 (S36 and lactose percentage), indicating that cows with higher milk production potential have a greater likelihood of remaining in the herd for a long time. In contrast, negative approximate genetic correlations were found between stayability traits and fat percentage, protein percentage, and SCS, ranging from −0.152 (S36 and protein rate) to −0.866 (S36 and SCS). Notably, the value of negative genetic correlations between stayability and SCS decreased as survival time increased, suggesting that cows completing more lactations tend to have lower somatic cell counts, thereby remaining in the herd for longer periods. In addition, the weak approximate genetic correlations were observed between stayability and urea nitrogen, ranging from −0.223 to 0.188.

### 3.5. Approximate Genetic Correlation Between Stayability and Conformation Traits

The approximate genetic correlations between stayability and conformation traits are shown in Table 4, and most conformation traits were positively correlated with stayability traits. Most body capacity traits, including body depth, chest width, and stature, showed negative correlations with stayability, ranging from −0.661 to −0.146, whereas loin strength was positively correlated, with estimates from 0.053 to 0.451, indicating that lower body capacity is associated with a greater likelihood of longer herd life. Positive approximate genetic correlations were also observed between stayability and most limb hoof traits (bone quality, foot angle, rear leg rear view, rear leg side view), and rear leg side view appears to have the strongest influence on stayability. Fore attachment, fore teat placement and rear attachment height showed positive correlations with stayability (0.054 to 0.875), while rear attachment width, rear teat placement, teat length, and angularity showed negative correlations (−0.718 to −0.067). This further indicates that cows with well-shaped udders, strong attachments, and moderate teat size tend to remain in the herd for a long time. In addition, the approximate genetic correlations between rump traits and stayability were negative, ranging from −0.914 to −0.237, suggesting that the extremely narrow rump trait is associated with a higher risk of early culling.

### 3.6. Approximate Genetic Correlation Between Stayability and Fertility Traits

The approximate genetic correlations between stayability and fertility traits are shown in Table 5. Stayability exhibited varying genetic associations with fertility traits. Positive approximate genetic correlations were found with heifer fertility traits (age at first calving, age at first service, interval from first to last inseminations in heifer), ranging from 0.081 to 0.875. Notably, these positive correlations tended to decline as survival time increased, further indicating that heifers with superior reproductive performance tend to have longer herd time. In contrast, days open, interval from calving to first insemination, interval from first to last inseminations in cow and birth weight displayed negative associations with stayability, with correlations ranging from −0.552 to −0.008. The conception rate of first insemination in cow showed a positive genetic correlation with stayability, whereas calving interval, calving ease, gestation length, calf survival presented correlations ranging from −0.680 to 0.408. Overall, these results suggest that long herd retention is typically linked to enhanced reproductive performance, especially traits associated with breeding success.

### 3.7. Approximate Genetic Correlation Between Stayability and Health Traits

The approximate genetic correlations between stayability and health traits are shown in Table 6. Udder health traits showed weak correlations with stayability, ranging from −0.397 to 0.312. Notably, these correlations tended to decline with increasing time in the herd. The approximate genetic correlations between stayability and reproductive disorders, locomotory diseases, digestive disorders, and metabolic disorders were low to moderate, ranging from −0.038 to 0.305, −0.454 to 0.209, −0.083 to 0.576, and −0.139 to 0.495, respectively. Locomotory diseases and abomasal displacement have the greatest impact on the herd time in dairy cows.

## 4. Discussion

### 4.1. Stayability Survival Analysis in Holstein Cattle

The benefit for cows begins from the second parity and reaches its highest level in the fifth parity. However, despite the higher benefits observed in later parities, cows’ survival decreases with time. In this study, the survival rate of Holstein cows showed a continuous decline, dropping from 0.23 at S36 to 0.08 at S84. In our previous study, we found that the cows used in the present research had an average age at first calving of 25 months [12]; therefore, the probability of our cows surviving to 5 years of age was 28%, which is substantially lower than the 38% to 43% survival rate to 5 years reported for Holstein cows by Garcia-Peniche et al. [20]. Currently, China’s dairy cattle industry is still in a phase of large-scale farming and relatively high individual milk yield per cow, which may lead to a higher rate of voluntary culling within herds. These factors could contribute to the poorer longevity observed in dairy cattle.

Our previous study found that reproductive disorders, digestive issues, and mastitis are the main reasons for culling [14]. Therefore, we further selected sterility and abortion (reproductive disorders), mastitis (udder health), and diarrhea, enteritis, and abomasal displacement (digestive disorders) for survival analysis. Abomasal displacement resulted in a rapid decline in survival probability and a shorter median survival time, which may be due to that dairy cows experience a state of negative energy balance during the 21 days before and after calving [21]. During this period, substantial changes occur in diet, metabolism, endocrine function, and immunity, leading to a higher incidence of abomasal displacement. Boulay et al. [22] reported that the probability of culling after surgery for abomasal displacement is as high as 12–17%. Consequently, this condition accelerates the culling process in dairy cows. In addition, Enteritis and mastitis are also important factors contributing to reduced survival probability. Enteritis impairs digestive and absorptive functions, resulting in nutritional deficiencies and physical weakness, whereas mastitis negatively affects both the health of the cow and the yield and quality of milk [23]. These conditions increase the risk of culling, thereby reducing survival probability. Infertility showed the slowest decline in survival probability and the longest median survival time, which may be attributable to culling decisions at the farm.

### 4.2. Genetic Parameters of Stayability Traits

The heritability of stayability traits in this study is 0.048–0.118, which is consistent with some previous studies [11], but lower than the 0.09–0.16 reported by Williams et al. [24] in Nellore cattle. The reason may be attributable to differences in population genetic backgrounds, statistical models, data size, and trait definitions. High genetic and phenotypic correlations were observed between adjacent stayability traits, which aligns with the studies of Hardie et al. [11]. In our study, phenotypic correlations ranged from 0.090 to 0.799, with the genetic correlations consistently exceeding phenotypic correlations. The trend of phenotypic correlations between stayability traits was consistent with genetic correlation, which is similar to reported studies [25]. The higher genetic correlation between stayability traits indicates that improving survival rate at one stage may also help increase survival rate at other stages, and this was beneficial for early direct selection of stayability traits in Holstein cattle.

### 4.3. Approximate Genetic Correlation Between Stayability and Production, SCS Traits

The low heritability of stayability traits suggests that improving them through direct selection alone would be slow. Therefore, combining them with indirect traits could achieve faster genetic gain. In dairy cattle, stayability is strongly influenced by milk production performance. In this study, stayability traits showed positive genetic correlations with milk yield, fat yield, and protein yield, but negative correlations with fat percentage and protein percentage. The directions of these genetic correlations were consistent with those reported in many previous studies [11,24,26], although the magnitudes were slightly higher in the present study. This is favorable, indicating that cows with higher genetic potential for milk, fat, and protein yields are more likely to remain in the herd. However, negative genetic correlations were also observed between production traits and lifespan score. Pritchard et al. [27] reported the genetic correlations between milk yield, protein yield, and fat yield and longevity scores in British dairy cattle, with coefficients ranging from −0.35 to −0.18. This may be explained by the increased metabolic and health stress associated with high milk production, which could reduce longevity in dairy cattle.

In our study, the approximate genetic correlations between stayability and lactose percentage ranged from low to high values. Miglior et al. [28] reported that cows with low lactose levels have a higher culling risk, whereas cows with high lactose levels have a lower culling risk. Similarly, Buckley et al. [29] found that a higher lactose percentage was associated with an increased pregnancy rate. These findings support the reliability of our results and indicate that lactose percentage may serve as an indirect indicator of stayability in dairy cattle. In contrast, the approximate genetic correlations between stayability and SCS were negative, and the values of genetic correlations decreased as the herd time increased. Hardie et al. [11] found that the genetic correlation between stayability and SCS ranged from −0.17 to −0.01, and Pritchard et al. [27] reported stronger negative correlations ranging from −0.56 to −0.45 between stayability and SCS. Although these results were in the same direction as ours, their magnitudes were smaller than those we observed. The discrepancies may be attributed to differences in population characteristics, analytical methods, and trait definitions. Additionally, animals with longer productive lifespans tend to have lower somatic cell counts, greater resistance to mastitis, and higher likelihoods of survival.

### 4.4. Approximate Genetic Correlation Between Stayability and Conformation Traits

Most body capacity traits, including body depth, chest width, and stature, showed negative correlations with stayability, ranging from −0.661 to −0.146. This is consistent with the results reported by Wasana et al. [30] and suggests that cows with relatively smaller body capacity are more likely to remain in the herd for longer periods. In contrast, Short et al. [31] found that the genetic correlations between body capacity and stayability ranged from 0 to 0.09, while Vukasinovic et al. [32] reported correlations ranging from −0.01 to 0.42. These differences may be attributed to variations in trait definitions, as well as differing emphasis placed on body capacity traits in breeding programs across countries. Weigel et al. [33] reported a positive genetic correlation between loin strength and stayability (0.31), which is similar to our results.

The health of the legs and hooves directly affects the locomotor ability and comfort of dairy cows. In this study, positive approximate genetic correlations were observed between stayability and most limb hoof traits, and rear legs’ side view appears to have the strongest influence on stayability. Similar results were reported by Van Doormal et al. [34] and Vukasinovic et al. [32] for Holstein cattle. Conversely, Rogers et al. [35] reported weak genetic correlations between leg and hoof traits and stayability traits, ranging from −0.01 to 0.20. Similarly, Harris et al. [36] used an alternative definition of longevity and also found weak genetic correlations with inconsistent directions, ranging from −0.14 to 0.16. Vukasinovic et al. [32] found that all udder and teat traits had positive genetic correlations with productive life, ranging from moderate to high. In contrast, in our study, most teat traits exhibited negative genetic correlations with stayability, suggesting that moderate teat length may be an important factor for a longer productive life, possibly due to its association with reduced susceptibility to injury. Comparable results were also reported by Gabriela Stefani et al. [37]. For rump traits, the approximate genetic correlation between stayability and rump ranged from −0.914 to −0.237, and the magnitude of the negative correlation decreased as survival time increased, indicating that the cow’s physiological traits gradually mature, thereby enhancing its resistance to culling. Berry et al. [38] reported that the rump can serve as an indicator for stayability. Cattle with a wider rump and a more favorable rump angle in cattle have been associated with reduced incidence of dystocia and decreased culling risk [39,40]. Therefore, selection for the rump trait is beneficial for improving the stayability of dairy cows.

### 4.5. Approximate Genetic Correlation Between Stayability and Fertility Traits

Reproductive disorders are a major reason for culling dairy cows in many countries. Therefore, fertility traits may serve as valuable auxiliary traits for the indirect selection of stayability traits, and combining stayability with fertility traits may help improve both simultaneously. In this study, we found positive approximate genetic correlations between heifer fertility traits and stayability, with the strongest correlations observed for age at first calving and age at first service. Similar findings have been reported in previous studies. Sewalem et al. analyzed factors affecting the longevity of Holstein, Jersey, and Ayrshire cattle and found that older heifers had a higher risk of culling than those that calved for the first time at 24 to 28 months of age. Additionally, Nilforooshan et al. reported that cows calving for the first time before 21 months of age faced a high risk of culling due to dystocia. The age at first service is greatly influenced by farm management decisions and economic factors, making it highly subjective. This may partly explain its relatively high genetic correlation with dairy cow stayability. Further studies with larger sample sizes are warranted to validate this conclusion.

Studying the genetic correlations between fertility and stayability in dairy cows is important, given the differences in additive genetic variance and heritability across parities. In this study, stayability showed negative genetic correlations with days open, interval from calving to first insemination, and interval from first to last insemination in cows. These results are consistent with the biological expectation that prolonged these traits generally reduces the likelihood of cows remaining in the herd for subsequent lactations, as delayed rebreeding can increase involuntary culling. Consistent with our results, Sewalem et al. [41] and Pinedo et al. [42] also reported associations between these reproductive traits and increased culling risk in dairy cows. In contrast, the conception rate of first insemination in cows showed a positive genetic correlation with stayability, and Hardie et al. [11] reported similar results. We also found that the approximate genetic correlation between stayability and gestation length, calving ease, and birth weight ranged from −0.680 to 0.066. Calving ease and gestation length appear to have relatively small effects on stayability in dairy cows based on the results of this study, but further research with larger datasets is warranted to validate this finding. Regarding calf-related factors, calf birth weight and survival showed a high negative genetic correlation with stayability. Sewalem et al. [41] found that cows producing calves of medium body size had a lower risk of culling. However, not all studies have reported consistent results; Jairath et al. [43] observed a positive genetic correlation between stayability and calf size. Such discrepancies may be attributable to differences in study populations, statistical methodologies, or other influencing factors considered. In addition, stayability was negatively correlated with calf survival, possibly because stillbirth can increase the incidence of mastitis and retained placenta [44]. Bicalho et al. [45] also reported that cows delivering stillborn calves had a significantly higher culling risk and prolonged days open.

### 4.6. Approximate Genetic Correlation Between Stayability and Health Traits

The approximate genetic correlation between stayability and udder health traits ranged from −0.397 to 0.312, which are comparable to the values reported in previous studies [27,46]. Shabalina et al. [6] reported genetic associations between stayability and mastitis during different lactation periods ranging from −0.77 to −0.26, which were of greater magnitude than our study. These results suggest that effective monitoring and management of udder health can contribute to prolonging the productive life of dairy cows. Shabalina et al. [6] also reported a strong negative genetic correlation (−0.98) between metabolic disorders and stayability traits. The discrepancy may be explained by differences in the incidence of digestive problems across study populations, as well as variations in the classification criteria used for recording health disorders.

Recent literature reported that the incidence of abomasal displacement in calving dairy cows in North America ranges from 3% to 7% [47,48,49]. Among cows undergoing corrective surgery, 12% to 17% are culled or die within 30 days post-operation [22]. In our study, abomasal displacement showed a higher genetic correlation with stayability, which is consistent with these studies. Therefore, strengthening early detection and prevention could enhance disease management and potentially improve both productivity and longevity in dairy herds. Overall, our results and previous reports emphasize that improving health traits (particularly those adversely affecting stayability) should be a priority in selection programs for stayability. However, the magnitude and direction of genetic correlations may vary across parities and environments; it is essential to develop herd-specific management practices and breeding strategies. Future studies should integrate genomic data to identify key genes influencing stayability and guide breeding programs that enhance cows’ health, stayability, and productivity.

## 5. Conclusions

Survival analysis revealed that cows with sterility had the longest median herd life (1670 days), whereas those affected by abomasal displacement had the shortest (1350 days) and a culling risk approximately 72% higher than that of the sterility group. The heritability for stayability traits was low, ranging from 0.048 to 0.118. The genetic correlations among stayability traits were positive, ranging from 0.382 to 0.975, and the phenotypic correlations among stayability traits were positive, ranging from 0.090 to 0.799, respectively. High genetic and phenotypic correlations were observed between adjacent stayability stages, indicating a strong shared genetic factor of these traits. Most of the approximate genetic correlations between stayability and routinely evaluated traits were low to moderate. This suggests that genetic improvement in stayability is feasible by including a selection index for stayability into routine genetic evaluations and accounting for the relationship among all traits. Particular attention should be paid to negative genetic relationships between traits. These findings provide valuable information for evaluating stayability in dairy cows and provide an important basis for developing scientifically sound breeding programs.

## Figures and Tables

**Table 1:** Descriptive statistics of stayability trait in Holstein cattle.

Trait (Units)	Definition	NTotal ^1^	Survival Probability
S36	the ability of cows to remain 36 months in herd after first calving	13,165	0.23
S42	the ability of cows to remain 42 months in herd after first calving	8982	0.19
S48	the ability of cows to remain 48 months in herd after first calving	6724	0.12
S54	the ability of cows to remain 54 months in herd after first calving	4472	0.08
S60	the ability of cows to remain 60 months in herd after first calving	3170	0.06
S72	the ability of cows to remain 72 months in herd after first calving	1352	0.024
S84	the ability of cows to remain 84 months in herd after first calving	430	0.08

^1^ NTotal = total number of observations.

**Table 2:** The heritability (diagonal), genetic correlation (above diagonal), and phenotypic correlation (below diagonal) for stayability in Holstein cattle.

Traits	S36	S42	S48	S54	S60	S72	S84
S36	0.048 (0.006)	0.910 (0.019)	0.836 (0.030)	0.783 (0.036)	0.735 (0.040)	0.623 (0.054)	0.382 (0.064)
S42	0.719 (0.0001)	0.063 (0.006)	0.975 (0.008)	0.939 (0.017)	0.882 (0.024)	0.735 (0.043)	0.472 (0.057)
S48	0.575 (0.0001)	0.799 (0.0001)	0.074 (0.007)	0.965 (0.009)	0.933 (0.016)	0.757 (0.038)	0.538 (0.050)
S54	0.428 (0.0001)	0.597 (0.0001)	0.750 (0.0001)	0.099 (0.007)	0.964 (0.008)	0.792 (0.031)	0.500 (0.045)
S60	0.338 (0.0001)	0.475 (0.0001)	0.598 (0.0001)	0.798 (0.0001)	0.115 (0.007)	0.881 (0.022)	0.568 (0.039)
S72	0.196 (0.0001)	0.281 (0.0001)	0.356 (0.0001)	0.478 (0.0001)	0.601 (0.0001)	0.088 (0.007)	0.785 (0.029)
S84	0.090 (0.001)	0.136 (0.001)	0.179 (0.0001)	0.243 (0.0001)	0.311 (0.0001)	0.532 (0.0001)	0.118 (0.008)

**Table 3:** Approximate genetic correlations of stayability traits with production traits in Holstein cattle.

Traits	S36	S42	S48	S54	S60	S72	S84
Milk yield	0.250 (0.007)	0.648 (0.005)	0.573 (0.005)	0.512 (0.004)	0.372 (0.005)	0.466 (0.005)	0.337 (0.005)
Fat percentage	−0.231 (0.007)	−0.339 (0.006)	−0.276 (0.006)	−0.329 (0.005)	−0.253 (0.005)	−0.221 (0.005)	−0.158 (0.005)
Protein percentage	−0.152 (0.007)	−0.472 (0.005)	−0.387 (0.005)	−0.386 (0.005)	−0.271 (0.005)	−0.344 (0.005)	−0.246 (0.005)
Fat yield	0.180 (0.007)	0.522 (0.005)	0.457 (0.005)	0.371 (0.005)	0.254 (0.005)	0.328 (0.005)	0.239 (0.005)
Protein yield	0.257 (0.007)	0.588 (0.005)	0.525 (0.005)	0.444 (0.005)	0.321 (0.005)	0.384 (0.005)	0.271 (0.005)
Lactose percentage	0.688 (0.006)	0.678 (0.005)	0.576 (0.005)	0.380 (0.005)	0.242 (0.006)	0.315 (0.006)	0.214 (0.006)
Urea nitrogen	−0.223 (0.010)	−0.031 (0.009)	0.188 (0.009)	0.044 (0.009)	0.045 (0.009)	0.181 (0.009)	−0.026 (0.009)
SCS	−0.866 (0.002)	−0.821 (0.004)	−0.605 (0.005)	−0.385 (0.005)	−0.279 (0.005)	−0.276 (0.005)	−0.212 (0.005)

**Table 4:** Approximate genetic correlations of stayability traits with conformation traits in Holstein cattle.

Traits	S36	S42	S48	S54	S60	S72	S84
Body depth	−0.494 (0.015)	−0.543 (0.013)	−0.474 (0.014)	−0.452 (0.014)	−0.442 (0.014)	−0.337 (0.015)	−0.146 (0.016)
Chest width	−0.347 (0.015)	−0.661 (0.011)	−0.446 (0.013)	−0.429 (0.013)	−0.382 (0.014)	−0.265 (0.014)	−0.159 (0.014)
Loin strength	0.362 (0.017)	0.451 (0.015)	0.38 (0.016)	0.2 (0.017)	0.122 (0.017)	0.053 (0.017)	0.21 (0.017)
Stature	−0.504 (0.010)	−0.414 (0.009)	−0.330 (0.009)	−0.34 (0.008)	−0.295 (0.008)	−0.354 (0.008)	−0.313 (0.008)
Bone quality	0.533 (0.020)	0.631 (0.018)	0.241 (0.023)	0.155 (0.154)	0.13 (0.023)	−0.094 (0.024)	0.195 (0.023)
Foot angle	0.032 (0.020)	0.159 (0.019)	0.125 (0.019)	0.132 (0.019)	0.173 (0.019)	0.020 (0.020)	0.017 (0.019)
Rear leg rear view	0.112 (0.118)	−0.057 (0.117)	0.075 (0.117)	0.204 (0.114)	0.155 (0.115)	0.04 (0.116)	0.111 (0.116)
Rear legs side view	0.662 (0.018)	0.826 (0.013)	0.510 (0.020)	0.428 (0.021)	0.314 (0.022)	−0.077 (0.024)	0.202 (0.023)
Heel depth	−0.115 (0.027)	−0.191 (0.027)	−0.161 (0.027)	−0.247 (0.026)	−0.190 (0.026)	−0.268 (0.026)	−0.161 (0.027)
Fore attachment	0.504 (0.012)	0.875 (0.006)	0.83 (0.007)	0.821 (0.007)	0.729 (0.008)	0.554 (0.010)	0.506 (0.010)
Fore teat placement	0.422 (0.03)	0.323 (0.031)	0.125 (0.033)	0.164 (0.033)	0.15 (0.033)	0.054 (0.033)	0.057 (0.033)
Median suspensory	0.379 (0.013)	0.121 (0.012)	−0.066 (0.012)	−0.013 (0.012)	−0.052 (0.012)	−0.088 (0.012)	−0.208 (0.012)
Rear attachment height	0.462 (0.018)	0.746 (0.013)	0.478 (0.018)	0.579 (0.016)	0.550 (0.017)	0.386 (0.019)	0.159 (0.020)
Rear attachment width	−0.342 (0.014)	−0.554 (0.011)	−0.718 (0.009)	−0.709 (0.009)	−0.632 (0.010)	−0.683 (0.010)	−0.627 (0.010)
Rear teat placement	−0.274 (0.013)	−0.252 (0.011)	−0.323 (0.010)	−0.237 (0.010)	−0.182 (0.010)	−0.067 (0.010)	−0.086 (0.010)
Teat length	−0.605 (0.014)	−0.697 (0.012)	−0.476 (0.015)	−0.432 (0.015)	−0.288 (0.016)	−0.276 (0.016)	−0.136 (0.016)
Udder depth	−0.134 (0.012)	0.168 (0.010)	0.395 (0.009)	0.377 (0.008)	0.392 (0.008)	0.459 (0.008)	0.38 (0.008)
Angularity	−0.607 (0.076)	−0.434 (0.086)	−0.294 (0.091)	−0.302 (0.090)	−0.21 (0.092)	−0.15 (0.093)	−0.176 (0.093)
Rump angle	−0.237 (0.013)	−0.462 (0.010)	−0.326 (0.010)	−0.358 (0.010)	−0.298 (0.010)	−0.298 (0.010)	−0.408 (0.010)
Rump width	−0.719 (0.010)	−0.914 (0.005)	−0.786 (0.007)	−0.695 (0.008)	−0.648 (0.008)	−0.511 (0.010)	−0.495 (0.009)

**Table 5:** Approximate genetic correlations of stayability traits with fertility traits in Holstein cattle.

Traits	S36	S42	S48	S54	S60	S72	S84
Age at first calving	0.875 (0.004)	0.834 (0.004)	0.699 (0.005)	0.515 (0.006)	0.394 (0.007)	0.207 (0.007)	0.127 (0.007)
Age at first service	0.737 (0.005)	0.663 (0.005)	0.457 (0.005)	0.339 (0.005)	0.255 (0.005)	0.213 (0.005)	0.158 (0.005)
Interval from first to last inseminations in heifer	0.081 (0.049)	0.148 (0.049)	0.187 (0.048)	0.211 (0.048)	0.2 (0.048)	0.184 (0.048)	0.063 (0.049)
Conception rate of first insemination in heifer	−0.017 (0.007)	0.256 (0.006)	0.225 (0.006)	0.023 (0.006)	−0.008 (0.006)	−0.141 (0.006)	−0.096 (0.006)
Calving interval	0.408 (0.048)	0.177 (0.052)	0.034 (0.053)	−0.215 (0.052)	−0.175 (0.052)	−0.236 (0.052)	−0.197 (0.052)
Days open	−0.243 (0.019)	−0.222 (0.019)	−0.510 (0.017)	−0.552 (0.016)	−0.510 (0.017)	0.415 (0.018)	−0.282 (0.019)
Interval from calving to first insemination	−0.008 (0.006)	−0.148 (0.006)	−0.346 (0.005)	−0.158 (0.005)	−0.204 (0.005)	−0.187 (0.005)	−0.193 (0.005)
Interval from first to last inseminations in cow	−0.409 (0.035)	−0.337 (0.036)	−0.518 (0.032)	−0.505 (0.033)	−0.397 (0.035)	−0.262 (0.036)	−0.15 (0.037)
Conception rate of first insemination in cow	0.259 (0.012)	0.368 (0.012)	0.764 (0.008)	0.673 (0.009)	0.364 (0.012)	0.077 (0.012)	0.117 (0.013)
Calving ease	0.141 (0.022)	0.113 (0.021)	−0.051 (0.021)	−0.066 (0.020)	−0.128 (0.019)	−0.156 (0.020)	−0.16 (0.019)
Gestation length	−0.106 (0.008)	−0.189 (0.007)	0.066 (0.007)	0.014 (0.006)	0.021 (0.006)	0.044 (0.007)	0.02 (0.006)
Calf survival	−0.238 (0.007)	−0.680 (0.005)	−0.255 (0.006)	−0.159 (0.006)	−0.066 (0.006)	0.187 (0.006)	0.001 (0.006)
Birth weight	−0.182 (0.009)	−0.251 (0.008)	−0.148 (0.007)	−0.183 (0.007)	−0.118 (0.007)	−0.046 (0.007)	−0.065 (0.007)

**Table 6:** Approximate genetic correlations of stayability traits with health traits in Holstein cattle.

Traits	S36	S42	S48	S54	S60	S72	S84
Udder health	0.075 (0.01)	0.077 (0.01)	−0.046 (0.01)	−0.0005 (0.01)	0.091 (0.01)	−0.142 (0.01)	−0.166 (0.01)
Mastitis	0.258 (0.06)	0.312 (0.07)	0.186 (0.06)	0.047 (0.06)	−0.083 (0.06)	−0.397 (0.05)	−0.317 (0.06)
Reproductive disorders	0.279 (0.08)	0.305 (0.08)	0.182 (0.08)	0.167 (0.08)	0.180 (0.08)	0.085 (0.08)	0.072 (0.08)
Gestation disorders and peripartum disorders	−0.035 (0.08)	0.042 (0.08)	−0.021 (0.08)	−0.038 (0.08)	0.048 (0.08)	0.028 (0.08)	−0.033 (0.08)
Irregular estrus cycle and sterility	0.251 (0.08)	0.293 (0.08)	0.180 (0.08)	0.148 (0.08)	0.111 (0.08)	0.024 (0.08)	0.126 (0.08)
Metritis	0.253 (0.08)	0.301 (0.08)	0.184 (0.08)	0.156 (0.08)	0.109 (0.08)	0.010 (0.08)	0.119 (0.08)
Locomotory diseases	−0.238 (0.08)	−0.303 (0.08)	−0.454 (0.08)	−0.371 (0.08)	−0.326 (0.08)	−0.193 (0.08)	−0.161 (0.09)
Claw diseases	−0.026 (0.07)	−0.309 (0.07)	−0.450 (0.07)	−0.451 (0.07)	−0.433 (0.07)	−0.274 (0.07)	−0.255 (0.07)
Laminitis complex	0.094 (0.09)	−0.014 (0.09)	0.209 (0.09)	0.025 (0.09)	−0.044 (0.09)	−0.095 (0.09)	0.032 (0.09)
Digestive disorder	−0.083 (0.07)	−0.072 (0.07)	0.018 (0.07)	−0.013 (0.07)	0.017 (0.07)	0.098 (0.07)	0.030 (0.07)
Abomasal displacement	−0.036 (0.04)	0.320 (0.03)	0.576 (0.007)	0.353 (0.03)	0.297 (0.03)	0.254 (0.03)	0.332 (0.03)
Metabolic disorders	−0.139 (0.02)	−0.040 (0.02)	0.231 (0.17)	0.171 (0.02)	0.118 (0.02)	0.234 (0.17)	0.185 (0.02)

## Data Availability

Dataset available on request from the authors. The raw data supporting the conclusions of this article will be made available by the authors on request.

## Supplementary Materials

The following supporting information can be downloaded at https://www.mdpi.com/article/10.3390/vetsci12111105/s1. Table S1: The number of data for each trait that used to analyze approximate genetic correlations.
