# Supplementary material for: Genetic Analysis of Stayability and its Relationships with Production, Conformation, Fertility and Health Traits in Holstein Cattle
Source: Vet Sci. 2025 Nov 19;12(11):1105. doi: 10.3390/vetsci12111105 (PMC12656929; doi:10.3390/vetsci12111105)
Supplement: Supplementary file 1 [file vetsci-12-01105-s001.zip › vetsci-3969229-supplementary.pdf]

Supplemental Table 1. The number of data for each trait that used to analyze approximate genetic correlations.

Traits	S36	S42	S48	S54	S60	S72	S84
Milk yield	18,169	26,025	30,164	36,482	39,240	34,223	39,840
Fat rate	18,136	25,964	30,080	36,349	39,050	34,113	39,631
Protein rate	18,169	26,025	30,166	36,485	39,244	34,227	39,844
Fat yield	18,135	25,963	30,081	36,344	39,024	34,107	39,604
Protein yield	18,151	25,994	30,123	36,409	39,134	34,164	39,721
Lactose percentage	13,948	20,309	23,429	28,388	30,614	26,549	31,055
Urea nitrogen	8,638	11,261	12,385	13,311	13,486	13090	13,497
SCS	2,587	13,269	13,269	13,269	13,269	13,269	13,269
Body depth	3,424	3,926	4,000	4,058	4,073	4,043	4,076
Chest width	3,756	4,423	4,543	4,619	4,637	4,600	4,641
Loin strength	3,037	3,325	3,377	3,420	3,427	3,407	3,427
Stature	6,810	9,507	10,960	12,973	13,827	12,235	13,979
Bone quality	1,730	1,769	1,776	1,797	1,798	1,790	1,798
Foot angle	2,462	2,586	2,605	2,633	2,636	2,624	2,636
Rear leg rear view	73	75	75	76	76	76	76
Rear legs side view	1,723	1761	1,768	1,789	1,790	1,782	1,790
Heel depth	1,341	1,363	1,370	1,380	1,381	1,379	1,381
Fore attachment	5,158	6,528	6,988	7,295	7,353	7,211	7,360
Fore teat placement	901	908	910	914	915	913	915
Median suspensory	5,060	6,360	6,782	7,038	7,090	6,964	7,096
Rear attachment height	2,347	2,449	2,463	2,489	2,490	2,480	2,490
Rear attachment width	4,473	5,467	5,719	5,858	5,890	5,813	5,895
Rear teat placement	5,878	8,080	9,094	10,170	10,435	9,802	10,474
Teat length	3,202	3,560	3,617	3,667	3,675	3,654	3,678
Udder depth	6,624	9,211	10,596	12,459	13,233	11,762	13,372
Rump angle	5,806	7,907	8,832	9,715	9,895	9,437	9,922
Rump width	5,497	7,198	7,832	8,309	8,401	8,175	8,415
Angularity	110	113	113	115	115	115	115
Age at first calving	14,753	17,574	18,506	19,224	19,367	19,054	19,387
Age at first service	20,444	27,249	31,943	37,903	40,215	35,973	40,714
Interval from first to last inseminations in heifer	412	416	419	421	421	421	421
Conception rate of first insemination in heifer	21,246	25,781	27,255	28,043	28,249	27,847	28,275
Calving interval	357	357	357	357	357	357	357
Days open	2,477	2,574	2,602	2,634	2,638	2,629	2,639
Interval from calving to first insemination	24,343	29,880	32,164	33,893	34,268	33,436	34,320
Interval from first to last inseminations in cow	697	701	701	701	701	701	701
Conception rate of first insemination in cow	6,177	6,244	6,252	6,257	6,261	6,259	6,261
Calving ease	1,980	2,218	2,346	2,561	2,668	2,493	2,691
Gestation length	14,937	18,900	21,038	23,878	25,185	22,811	25,444
Calf survival	21,449	24,124	24,780	25,278	25,426	25,155	25,447
Birth weight	12,376	16,025	18,096	20,634	21,727	19,753	21,938
Udder health	5,595	5,726	5,743	5,747	5,750	5.746	5,745
Mastitis	289	287	289	289	289	288	289
Reproductive disorders	162	162	162	162	162	162	162
Gestation disorders and peripartum disorders	146	146	146	146	146	146	146
Irregular estrus cycle and sterility	155	155	155	155	155	155	155

Metritis	159	159	159	159	159	159	159
Locomotory diseases	137	137	137	137	137	137	137
Claw diseases	175	175	175	175	175	175	175
Laminitis complex	116	116	116	117	116	116	116
Digestive disorder	207	207	207	207	207	207	207
Abomasal displacement	625	797	857	896	917	917	916
Metabolic disorders	3,270	3,326	3,333	3,336	3,338	3,337	3,335
Ketosis	3,477	3,546	3,557	3,559	3,562	3,560	3,558
